# INSIG-2 promoter polymorphism and obesity related phenotypes: association study in 1428 members of 248 families

**DOI:** 10.1186/1471-2350-7-83

**Published:** 2006-11-30

**Authors:** Darroch H Hall, Thahira Rahman, Peter J Avery, Bernard Keavney

**Affiliations:** 1 Institute of Human Genetics (DH, TR, BK), and School of Mathematics and Statistics (PJA), Newcastle University, UK

## Abstract

**Background:**

Obesity is a major public health problem. Body mass index (BMI) is a highly heritable phenotype but robust associations of genetic polymorphisms to BMI or other obesity-related phenotypes have been difficult to establish. Recently a large genetic association study showed evidence for association of the single nucleotide polymorphism (SNP) rs7566605, which lies 10 Kb 5' to the first exon of the insulin-induced gene 2 (INSIG-2), with obesity in several cohorts. We tested this polymorphism for association with body mass related phenotypes in a large family study whose mean BMI was consistent with moderate overweight.

**Methods:**

We studied 1428 members of 248 British Caucasian families who had been ascertained through a proband with hypertension. We measured BMI, waist and hip circumference, and plasma levels of leptin. We genotyped the rs7566605 SNP using a restriction fragment length polymorphism assay, and carried out a family-based association test for quantitative traits related to obesity using the statistical programs MERLIN and QTDT.

**Results:**

We observed no significant association between genotype at rs7566605 and covariate-adjusted (for age, sex, alcohol consumption, smoking and exercise habit) log-transformed BMI, waist measurement, hip measurement, waist-to-hip ratio, or plasma levels of leptin.

**Conclusion:**

There was no association between genotype at rs7566605 and obesity-related phenotypes in this British Caucasian population. These families were in general moderately overweight, few members being severely obese. Our result indicates that this polymorphism has little if any effect on BMI within the normal to moderately overweight range. The effects of this polymorphism on body mass may be restricted to those already predisposed to at least moderate obesity as a result of environmental factors and other predisposing genotypes.

## Background

Obesity is an increasingly prevalent public health problem worldwide [1]. Obesity is a major risk factor for the development of hypertension, diabetes, coronary heart disease and stroke [2]. Secular trends towards overweight indicate the strong influence of lifestyle on the risk of obesity; nevertheless multiple studies assessing heritability within families have shown evidence for significant genetic influences on body mass index [3,4]. Identification of these genetic determinants has thus far proved difficult. Recently, Herbert and colleagues identified, following a genome-wide association study involving 86,604 SNPs, one SNP (rs7566605) situated 10 Kb 5' to the insulin-induced gene 2 (INSIG-2) which showed strong evidence for association with body mass index in multiple cohorts (OR for obesity 1.22 [95% CI 1.05–1.42]; p = 0.008 in meta-analysis of 6 cohorts). Homozygotes for the minor C allele (around 10% of the population) had BMI about 1 Kg/m^2 ^higher than did homozygotes for the major G allele or GC heterozygotes [5]. That study tended to show the strongest evidence for association in cohorts selected for severe obesity; in one of the cohorts tested (the Nurses' Health Study cohort) whose mean BMI was more representative of the majority of overweight patients there was no association between the SNP and obesity. Obesity is a major risk factor for hypertension, but most hypertensives are moderately, rather than severely overweight [6]. We therefore tested the association of rs7566605 with body mass index in a large family collection, selected through a proband with hypertension, which we have previously used successfully to identify SNPs associated with obesity related phenotypes.

## Methods

### Subject collection and phenotyping

The collection strategy of this family study has been previously described [7]. Briefly, families were ascertained through a proband with essential hypertension. In order to be suitable for the study, families were required to consist of at least three siblings clinically assessable for blood pressure if at least one parent of the sibship was available to give blood for DNA analysis, and to consist of at least four assessable siblings if no parent was available. Where members of the sibship were found to be hypertensive, families were extended and the spouses and offspring of hypertensive sibs collected. Thus, the majority of the individuals in the family collection have blood pressures within the normal range, and the family collection includes some extended families, though most are nuclear families. A full medical and lifestyle history was taken. Subjects underwent automated ambulatory blood pressure monitoring for 24 hours (A&D TM2421 monitor) with readings taken half-hourly by day and hourly by night. Anthropometric measurements including height, weight, and waist and hip circumferences were made (waist measured at the natural waist, and hip measured at the level of the greater trochanters). Body mass index was calculated as the weight (in Kg) divided by the square of height (in m). 1428 individuals from 248 families participated in the study. The study was approved by the appropriate local Ethics Committee. All participants gave informed consent to participate in the study.

### Laboratory methods

Plasma leptin was measured using a commercially available ELISA kit (Linco Research, St. Charles, MO) with sensitivity 0.5 ng/ml, intra-assay precision 2.6–4.6% and inter-assay precision 2.6–6.2%. The rs7566605 SNP was genotyped by PCR using forward and reverse primers 5'-CCC TCC AAT ACC CCA TCG GA-3' and 5'-GGG AAT CGA GAG CTA AGG AT-3', respectively. Each 15 μl PCR reaction mixture consisted of 25 ng genomic DNA, 0.2 μM of each primer, 0.2 mM dNTPs, 2.5 mM MgCl_2_, 1 × HotStarTaq reaction buffer, and 0.2 units of HotStarTaq DNA polymerase (QIAGEN, Crawley, UK). The amplification procedure consisted of initial denaturation at 95°C for 15 min, 40 cycles of denaturation at 95°C for 20 s, annealing at 52°C for 30 s, and extension at 72°C for 1 min, followed by a final extension at 72°C for 3 min. The 182 bp PCR amplicon was digested with *Mbo *I restriction enzyme (Promega, Madison, USA). The 20 μl reaction volumes contained 4 μl PCR product, 1 × reaction buffer C, 0.1 mg/ml Acetylated BSA, 2 units *Mbo *I. Digests were carried out at 37°C for 15 hours to ensure complete digestion of the PCR product. Fragments were separated by electrophoresis on 3% agarose gels. There is no *Mbo *I restriction site in the G allele amplicon, yielding a band of 182 bp. The C allele amplicon has one *Mbo *I restriction site, resulting in two restriction fragments of 67 bp and 115 bp. Reference individuals of known genotype were included in every run. 10% of samples were re-genotyped and no discordance between genotype calls was observed, yielding an estimated genotyping error rate of <1%. Genotypes were successfully scored on over 95% of individuals with DNA available.

### Statistical analysis

Phenotypes of interest were examined for Normality and log-transformed in the case of body mass index (BMI) and plasma leptin to achieve an approximately Normal distribution. The distribution of waist-hip ratio did not depart significantly from Normality. Significant covariates of these phenotypes were then determined by linear regression using MINITAB; the adjusted values from these regressions were standardised (so each phenotype has a mean of zero and a standard deviation of 1) and used in the genetic analyses. The covariates age, sex, smoking (graded current/former/never), presence of cardiovascular medications, alcohol consumption (in units per week), exercise habit (graded none regular/1 or 2 times per week/three or more times per week) were considered. Mendelian inheritance of all genotypes and correspondence of genotype frequencies to Hardy-Weinberg proportions was checked using PEDSTATS [8]. Association between genotypes and the adjusted phenotypes was assessed in the families by calculating identity-by-descent vectors for each individual using MERLIN [9] followed by variance components analysis considering BMI and waist-hip ratio as quantitative traits using QTDT [10]. Models incorporated a dominance parameter since the original observation had been a recessive effect of the C allele. We also carried out TDT analyses in which those individuals with age- and sex-adjusted BMI >30 (N = 206) were designated affected and those with BMI < 25 (N = 733) unaffected, those with BMI between 25 and 30 being designated unknown.

## Results

Characteristics of the study subjects are shown in Table 1. 60% of families comprised between 4 and 6 genotyped members. Median values for BMI, WHR and plasma leptin lie towards the upper end of the normal range for an unselected UK Caucasian population [11], while daytime systolic and diastolic blood pressures would be consistent with selection of these families through a hypertensive proband. Correction for age, sex, alcohol consumption, smoking and habitual exercise accounted for about 15% of the total variability in log BMI, and 46–49% of the total variability in WHR and log plasma leptin level. The heritability of the obesity phenotypes after correction was 35% for log BMI, 28% for WHR and 36% for log plasma leptin (all p < 0.0001).

**Table 1:** Characteristics of the study population

Variable	n	min	LQ	median	UQ	max	R^2†^
Age (yr)	1425*	18.7	35.7	50.9	60.9	90.7	-
BMI (kg/m^2^)	1402	16.7	23.1	25.4	28.2	51.8	15.2
WHR	1357	0.56	0.78	0.85	0.91	1.22	48.7
Plasma Leptin (ng/μl)	1319	1.1	4.6	8.6	15.3	116.6	46.4
Daytime systolic BP (mmHg)	958	94.2	121.1	131	144.1	214.0	20.4
Daytime diastolic BP (mmHg)	958	54.0	72.0	78.6	88.0	119.9	17.9

* of which 52.4% were female and 36.1% were classified as hypertensive.

^† ^After correction for age, sex, alcohol consumption, smoking behaviour, and exercise habit.

LQ = lower quartile, UQ = upper quartile

**Figure 1:**
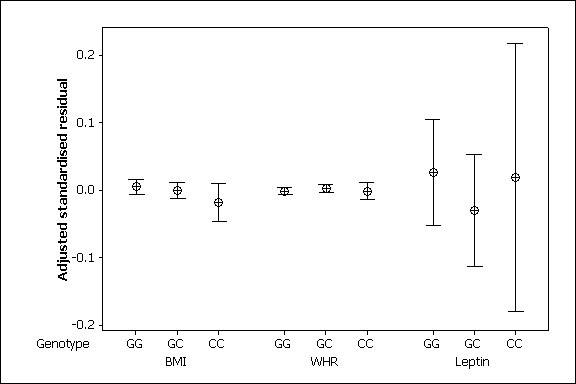
Means and 95% CIs of adjusted standardised log BMI, waist-hip ratio and log Leptin grouped by rs7566605 genotype. Numbers in the three genotype groups are GG 689, GC 570, CC 113.

Genotype frequencies at rs7566605 were in correspondence with Hardy-Weinberg proportions (p > 0.1). The frequency of the G allele in this population was 0.69 and of the C allele 0.31, which is in good agreement with the Caucasian populations genotyped in the study by Herbert et al [5]. The polymorphism had 40% heterozygosity. The mean values of BMI, waist-hip ratio and plasma leptin in each of the three genotype groups are shown in Table 2. There was no significant association between genotype and covariate-adjusted, log-transformed BMI measured as a continuous variable either under the recessive model (CC versus GG and GC; p > 0.10) found to be significant in the study by Herbert et al., under a codominant model (CC versus CG versus GG; p > 0.10), or under a dominant model (CC and CG versus GG; p > 0.10). Neither was there evidence of association between genotype and obesity affection status (defined as BMI > 30, with control status BMI < 25) under any model. There was no significant association between genotype and the obesity-related phenotypes WHR or log plasma leptin level as continuous variables (p > 0.1 for all comparisons; Figure 1).

**Table 2:** Genotype-phenotype association at rs7566605

	rs756605 genotype		
	G/G	G/C	C/C

N	689	570	113
BMI Kg/m^2^	26.132 (0.167)	25.944 (0.179)	25.626 (0.420)
Waist-hip ratio	0.853 (0.004)	0.858 (0.004)	0.848 (0.009)
Plasma Leptin ng/μL	12.53 (0.51)	11.92 (0.51)	12.70 (1.20)

Figures are means (standard errors)

## Discussion

This study of 1428 individuals from 248 families with median BMI 25.4 (indicating that about half the cohort were overweight) did not show any association between the CC genotype of the rs7566605 SNP, situated some 10 Kb 5' to the INSIG-2 gene, and body mass index, waist-hip ratio, or plasma leptin levels as quantitative traits. Nor was there any association between genotype and obesity when considered as an affection status, although our study contained relatively few such individuals. These results suggest that the effect of the rs7566605 SNP on BMI within the range encompassing normal and overweight is at most small, despite the evidence from a previous study that the CC genotype at this SNP may be a significant risk factor for obesity (classified as BMI > 30) [5].

It is possible that this study did not have sufficient power to replicate the association described by Herbert et al. The "winner's curse" phenomenon has been invoked to explain the frequently observed phenomenon in genetic epidemiology that first studies reporting a novel association very frequently show more extreme odds ratios than subsequent replication studies [12]. If the estimated OR for obesity of 1.22 in the study by Herbert et al. is in fact an upwardly biased estimate of the true association, this might explain our negative result. Also, families in the present study were not selected for obesity, which would tend to decrease their power to replicate the association. However, the present family collection has previously detected quantitative genetic effects contributing less than 5% of the total population variability in several traits (including obesity-related phenotypes) for which the families were not selected; these associations have themselves been replicated in other studies [13-19]. Moreover, the original test cohort showing a significant association (from the Framingham Heart Study) in the paper by Herbert et al. were unselected for obesity indeed, the BMI distribution was similar to that in the present study – and that Framingham cohort was smaller than the present study. Lack of power is therefore an unlikely explanation for non-replication.

The families in the present study were selected through a hypertensive proband; selection bias could in theory be the reason for non-replication. However, since we did not select against overweight, since hypertension is not thought to directly cause obesity, and since two-thirds of the family members were non-hypertensive, this seems unlikely. We only typed the rs7566605 SNP in these analyses. If the previously observed association arose through linkage disequilibrium (LD) between rs7566605 and a neighbouring causative SNP, and if that LD between alleles at rs7566605 and that SNP were weaker in our population than the populations studied by Herbert et al., that might account for the negative result. In the absence of additional genotyping data at neighbouring SNPs, we cannot entirely rule out this possibility. However, the families studied here have previously shown very similar haplotype frequencies and LD patterns to other European Caucasian cohorts and HapMap data on Caucasian families at multiple loci, so this also seems unlikely to explain our negative findings.

Several of the populations tested by Herbert et al. had been selected for extreme and/or childhood onset obesity [5]. No association was observed in the 2726 participants in the Nurses' Health Study cohort that were genotyped in that report; members of that cohort had a significantly lower mean BMI than the other populations studied, although about half were overweight (median BMI 24.89; IQR 22.35–28.43), as in the present study. Our findings, considered together with the negative result in the Nurses' Health Study cohort obtained by Herbert et al., suggest that that the effects of the rs7566605 SNP on body mass may be apparent only in individuals who are already predisposed to significant obesity for other genetic and environmental reasons. Such a notion would be in keeping with what is known about the function of the INSIG-2 gene. Further studies in overweight populations will be necessary to resolve this issue, and to detect such interacting factors.

## Conclusion

The majority of people at risk from atheromatous cardiovascular disease and diabetes have BMIs in the range 25–30. The negative results of the present study pertain to such a moderately overweight cohort who are at additional risk of cardiovascular disease by virtue of a familial tendency to hypertension. They suggest that the public health implications of the previously described genetic association between the rs7566605 SNP and BMI may be at most moderate.

## Competing interests

The author(s) declare that they have no competing interests.

## Authors' contributions

DH designed the genotyping assay, genotyped families, and analysed data. TR genotyped families. PJA analysed and interpreted data. BK designed the study, collected families, analysed and interpreted data and drafted the manuscript. All authors contributed to the final critical revision of the manuscript.

## Pre-publication history

The pre-publication history for this paper can be accessed here:


